# Bipolar disorder in the digital age: new tools for the same illness

**DOI:** 10.1186/s40345-016-0065-1

**Published:** 2016-11-17

**Authors:** John Torous, Paul Summergrad, S. Nassir Ghaemi

**Affiliations:** 1 Department of Psychiatry, Beth Israel Deaconess Medical Center, Harvard Medical School, 330 Brookline Ave, Boston, MA 02446 USA; 2 Division of Clinical Informatics, Beth Israel Deaconess Medical Center, Harvard Medical School, Boston, MA USA; 3 Department of Psychiatry, Tufts Medical Center, Tufts University, Boston, MA USA

**Keywords:** Bipolar, Technology, Apps, Big data, Nosology

## Abstract

“Nothing is more difficult than to ascertain the length of time that a maniacal patient can exist without sleep.”—Dr. Sutherland (Br J Psychiatry 7(37):1–19, 1861). Dr. Sutherland’s patient was suffering from an acute manic episode, which today is called bipolar illness. 150 years later, we continue to struggle with the same challenges in ascertaining accurate symptoms from patients. In era of new digital tools, the quantified self-movement, and precision medicine, we can ask the question: Can we advance understanding and treatment for bipolar illness beyond asking the same questions as in 1861?

## Notecards

The greatest breakthrough in understanding this disease did not require any technology at all. Dr. Sutherland would have offered his patient the only diagnosis that existed at the time: insanity. A few decades later, the German psychiatrist Emil Kraepelin began a series of careful observations utilizing notecards to longitudinally assess symptoms and outcomes in insane hospitalized patients. Years of carefully collected data revealed at least two major different courses of illness, with one group remaining delusional all the time, and never improving, while another had time-limited episodes of delusions, with eventual recovery, followed by later relapse. The resulting conceptualization of manic-depressive insanity (MDI), as Kraepelin defined it, was distinguished from dementia praecox (later revised by others to “schizophrenia”), thus breaking down the broad label of insanity into two different illnesses (Torous and Keshavan 2014). This distinction continues to have considerable clinical salience, despite heavy criticism over the past century. In the last 40 years, the broad manic-depressive concept was redefined in DSM-III in 1980 to a narrower bipolar disorder concept and a much broader major depressive disorder (MDD) concept (Decker 2007). Bipolar illness, as the term is now used in the DSM-5 (Angst 2013), is not the same thing as MDI, but is rather smaller part of the latter. Contrary to common belief, Kraepelin’s original view of MDI did not involve “classic episodic” bipolar disorder, but rather the reverse: he held that mixed states were the most common mood state, and polarity was irrelevant to diagnosis: what are now called “unipolar” depressive episodes were viewed by Kraepelin as part of MDI (Ghaemi and Dalley 2014). Nonetheless, much of Kraepelin’s observations have been retained, though revised, in the bipolar and MDD constructs (Duffy et al. 2016).

Kraepelin’s innovation was based on a simple kind of technology, the use of note cards and the application of basic statistical enumeration: counting. Such great success with such basic methods suggests that other technology and methods might have a larger potential. A particular question is what may be the impact of today’s digital devices which are capable of measuring much more data, and of different types than Kraepelin or other more recent researchers could ever have imagined.

## Digital technology

Despite this potential, initial studies with digital technologies for monitoring or augmented diagnosis in bipolar illness have not yielded anything near the profound results that Kraepelin achieved with his simple notecards. Actigraphy research, broadly defined as the use of non-invasive tools to measure human behavior and patterns, has suggested that patients with bipolar illness are willing to wear custom watches to detect sleep and activity patterns, and even wear t-shirts with embedded cardiac sensors (Migliorini et al. 2011). But nothing near a diagnostic breakthrough is likely to occur because of adherence issues. Considering actigraphy for patients with bipolar illness, one study observed that the “system requires a large number of sensors and devices… result[ing] in user rejection of the system altogether” (Prociow et al. 2012). Kraepelin was able to obtain long-term careful observation of patients because they were confined to state hospitals for years. Today, hospitalizations are very brief, usually a week or so in length, and patients treated outside hospitals, in clinical settings, are both unlikely to wear special sensors or devices for extended periods of time, and may experience such techniques as an intrusion on their privacy. It appears that actigraphy will not be able to provide valuable long-term longitudinal data needed to advance understanding of bipolar illness.

The recent rise of smartphones has rekindled interest in actigraphy, especially for bipolar illness, and there is hope that these devices and their related apps may finally yield valuable clinical information that may aid in diagnosis and monitoring. Like everyone else, people with bipolar illness increasingly own and use smartphones and other connected devices. Smartphones and many wearables are able to collect data, such as location via GPS sensors and voice data from conversations and social metrics, via call and text logs—all without the user taking any additional action (Faurholt-Jepsen et al. 2016; Abdullah et al. 2016). These features raise the possibility that adherence to data collection will be less of obstacle as with actigraphy. Yet early research using smartphones to record symptoms of bipolar illness has been equivocal. This is likely in part due to the early nature of this research with many studies small in sample size and not randomized or even controlled (Kaplan and Stone 2013). Detecting state transitions to mania and depression may require large-scale longitudinal studies. Some symptoms should be easier to assess than others. Sutherland’s focus on sleep, which is among the most objective of manic symptoms, should be the easiest question to solve. Smartphones already provide extensive data on sleep patterns. There is promise that they can characterize the nature of manic insomnia, if we apply them to systematic research on bipolar illness. But other aspects of the manic syndrome seem to be more difficult to explore, with new data streams that are complex and often difficult to interpret (Monteith et al. 2015; Faurholt-Jepsen et al. 2015).

How can it be that in this age where we can collect so much information from patients, more than one million data points per day from smartphones (Torous et al. 2016; Monteith et al. 2016), we still cannot rival the results and insights of Kraepelin’s notecards?

## Back to Kraepelin

There is no simple answer to this question, but two features of Kraepelin’s work may suggest possible solutions. First, he did not set out to determine how well his notecard data could match the then current concept of insanity. Rather than trying to fit his data to the conventional nosology of his time, he let the data guide him towards a new nosology. Second, he made his observations over years, in some cases decades, before drawing conclusions about the meaning of the data. Today, studies are usually short in duration, often only a few weeks and rarely longer than a year. Grant funders and researchers today are impatient; the concept of long-term outcome is rarely funded or studied in psychiatry, with the longest randomized clinical trials routinely being one year at most. There is no inherent reason why this should be the case. We note that cardiology studies, even in complex double-blind randomized trials, commonly are conducted for five years or longer. Yet we make diagnoses that last a lifetime, and we give medications for decades.

These observations suggest a new insight: Perhaps our digital tools are fine, even excellent in the purely technical sense. Our problem is that we are not applying them effectively or thoughtfully.

To follow Kraepelin’s example, we would need the courage, while starting with clinically defined populations to allow the longitudinal data to guide diagnostic definitions, rather than the other way around. We also would need to slow down the process, and obtain data for longer periods, rather than weeks or months, and from much larger groups of patients and controls, before drawing meaningful conclusions. New statistical methods are also need to make sense of the large amount of data collected (Torous et al. 2015). We also need to be thoughtful about how we employ these technologies. The foundation of health is trust, and thus there must be more emphasis on transparency and data security. More focus on the user perspective and design will likely increase adherence and acceptance.

We have the technology. Now we have find methods to collect data which can be acceptable to patients, family members, IRBs, and the general public, especially considering the continued stigma associated with psychiatric illnesses, and the not insubstantial concerns that have been raised about the safety and privacy of digital data. Maintaining confidentiality of such vast amounts of longitudinal data will require more than just digital safeguards with a need to also focus training on people, processes, and regulations surrounding such sensitive information (Armontrout et al. 2016). It will also require us to be open, as in all good research to where the findings take us. This requires a scientific attitude of self-criticism and openness to change, as well as demonstrating the superiority or at least the non-inferiority of such data in assessing and caring for real-world patients.

It is time to make use of our powerful new technologies, but to do so, we have to prepare our minds to use those technologies most effectively, and to think through carefully both the ethical and methodological issues at hand. It will also require funding streams that are not driven by quarter-to-quarter market results. In the end, it may also be that the mentality of the note taker matters as much as the use of the notecard.

## References

[CR1] Abdullah S, Matthews M, Frank E, Doherty G, Gay G, Choudhury T (2016). Automatic detection of social rhythms in bipolar disorder. J Am Med Inform Assoc.

[CR2] Angst J (2013). Bipolor disorders in the DSM-5: strengths, problems, and perspectives. Int J Bipolar Disord..

[CR3] Armontrout J, Torous J, Fisher M, Drogin E, Gutheil T (2016). Mobile mental health: navigating new rules and regulations for digital tools. Curr Psychiatry Rep.

[CR4] Decker HS (2007). How kraepelinian was Kraepelin? How kraepelinian are the neo-Kraepelinians?–from Emil Kraepelin to DSM-III. Hist Psychiatry.

[CR5] Duffy A, Malhi GS, Grof P. Do the trajectories of bipolar disorder and schizophrenia follow a universal staging model? Can J Psychiatry. 2016. pii:0706743716649189. (**epub ahead of print**) 10.1177/0706743716649189 PMC5298521 27310243

[CR6] Faurholt-Jepsen M, Vinberg M, Frost M, Christensen EM, Bardram JE, Kessing LV (2015). Smartphone data as an electronic biomarker of illness activity in bipolar disorder. Bipolar Disord.

[CR7] Faurholt-jepsen M, Busk J, Frost M (2016). Voice analysis as an objective state marker in bipolar disorder. Transl Psychiatry.

[CR8] Ghaemi SN, Dalley S (2014). The bipolar spectrum: conceptions and misconceptions. Aust NZ J Psychiatry.

[CR9] Kaplan RM, Stone AA (2013). Bringing the laboratory and clinic to the community: mobile technologies for health promotion and disease prevention a. Annu Rev Psychol.

[CR10] Migliorini M, Mendez MO, Bianchi AM (2011). Study of heart rate variability in bipolar disorder: linear and non-linear parameters during sleep. Front Neuroeng.

[CR11] Monteith S, Glenn T, Geddes J, Bauer M (2015). Big data are coming to psychiatry: a general introduction. Int J Bipolar Disord.

[CR12] Monteith S, Glenn T, Geddes J, Whybrow P, Bauer M (2016). Big data for bipolar disorder. Int J Bipolar Disord.

[CR13] Prociow P, Wac K, Crowe J (2012). Mobile psychiatry: towards improving the care for bipolar disorder. Int J Mental Health Syst.

[CR14] Sutherland AJ (1861). Croonian lectures. On the pathology, morbid anatomy, and treatment of insanity, delivered at the royal College of physicians, London, 1858. Br J Psychiatry.

[CR15] Torous J, Keshavan M (2014). The future of psychoses as seen from the history of its evolution. Curr Behav Neurosci Rep.

[CR16] Torous J, Staples P, Onnela JP (2015). Realizing the potential of mobile mental health: new methods for new data in psychiatry. Curr Psychiatry Rep.

[CR17] Torous J, Lorme J, Kiang M, Onnela JP (2016). New tools for new research in psychiatry: a scalable and customizable platform to empower data driven smartphone research. JMIR Mental Health..

